# Does reproductive isolation reflect the segregation of color forms in *Spiranthes sinensis* (Pers.) Ames complex (Orchidaceae) in the Chinese Himalayas?

**DOI:** 10.1002/ece3.4067

**Published:** 2018-04-27

**Authors:** Zhi‐Bin Tao, Zong‐Xin Ren, Peter Bernhardt, Huan Liang, Hai‐Dong Li, Yan‐Hui Zhao, Hong Wang, De‐Zhu Li

**Affiliations:** ^1^ Key Laboratory for Plant Diversity and Biogeography of East Asia Kunming Institute of Botany Chinese Academy of Sciences Kunming China; ^2^ Kunming College of Life Sciences University of Chinese Academy of Sciences Kunming China; ^3^ Department of Biology St. Louis University St. Louis Missouri; ^4^ Germplasm Bank of Wild Species Kunming Institute of Botany Chinese Academy of Sciences Kunming China

**Keywords:** bee pollinators, floral color, floral scent, phylogenetics, reproductive isolation, *Spiranthes sinensis*

## Abstract

Isolation between species, or taxa sharing a common lineage, depends primarily on the relative strengths of various reproductive barriers. Previous studies on reproductive isolation between orchids emphasized mechanical and ethological barriers in flowers of species showing food and/or sexual mimicry. In this study, we investigated and quantified a series of prepollination and postpollination barriers between pink and white forms of *Spiranthes sinensis* sl, a nectar‐secreting complex. We generated ML trees based on *trn*S‐G and *mat*K to explore phylogenetic relationships in this species complex. *Spiranthes sinensis* sl segregated from some other congeners, but the white form constituted a distinct clade in relation to the pink form. The white form secreted 2‐Phenylethanol as it is a single‐scent compound and was pollinated almost exclusively by native, large‐bodied *Apis cerana* and *Bombus* species (Apidae). *Apis cerana* showed a high floral constancy to this form. The scentless, pink form was pollinated primarily by smaller bees in the genera *Ceratina* (Apidae), and members of the family Halictidae, with infrequent visits by *A. cerana* and *Bombus* species. Fruit set and the production of large embryos following interform pollination treatments were significantly lower compared to intraform pollination results for the white form. Our results suggested that pollinator isolation, based on color and scent cues, may result in greater floral constancy in white populations when both forms are sympatric as two different, guilds of pollinators forage selectively preventing or reducing prospective gene flow. Postpollination barriers appear weaker than prepollination barriers but they also play a role in interform isolation, especially in the white form. Our findings suggest that floral color forms in *S. sinensis* do not represent an unbalanced polymorphism. Interpretations of the evolutionary status of these forms are discussed.

## 1 INTRODUCTION

The evolution and maintenance of discrete species or related lineage integrity depend largely on the effectiveness of various modes of interspecific reproductive isolation (Coyne & Orr, 2004; Grant, 1981; Mayr, 1992). The identification and analyses of these barriers can facilitate our understanding of the process of speciation and may help clarify earlier taxonomic treatments (Lowry, Modliszewski, Wright, Wu, & Willis, 2008; Lowry, Rockwood, & Willis, 2008; Widmer, Lexer, & Cozzolino, 2009). Isolation barriers in flowering plants are usually subdivided according to whether they are most effective at the prepollination stage (i.e., distribution, flowering periods, floral morphometrics, different pollinator guilds) or at the postpollination stage (i.e., interspecific incompatibility, embryonic hybrid inviability, F_1_ sterility and see Lowry, Modliszewski et al., 2008; Lowry, Rockwood et al., 2008).

Within a lineage containing several plant species, reproductive isolation is usually maintained by the employment of a suite of reproductive barriers instead of just one isolating mechanism (Baack, Melo, Rieseberg, & Ortiz‐Barrientos, 2015; Lowry, Modliszewski et al., 2008; Lowry, Rockwood et al., 2008; Widmer et al., 2009). In particular, it is necessary to record the chronology and rate at which different barriers emerge over a life history to better contrast the roles of ecological vs. molecular interactions responsible for reducing gene flow among sympatric populations (Baack et al., 2015; Cozzolino & Scopece, 2008; Lowry, Modliszewski et al., 2008; Lowry, Rockwood et al., 2008; Ramsey, Bradshaw, & Schemske, 2003; Widmer et al., 2009). Based on a comprehensive meta‐analysis of 19 plant species pairs, Lowry, Modliszewski et al. (2008) and Lowry, Rockwood et al. (2008) suggested that prezygotic barriers contributed more to reproductive isolation than postzygotic barriers. This hypothesis was supported in other studies (Lowry, Rockwood et al., 2008; Pellegrino, Bellusci, & Musacchio, 2010; Sobel & Streisfeld, 2015; Xu et al., 2011), but there are important exceptions in some lineages (Borba, Shepherd, & Semir, 2001; Chen, Luo, & Zhang, 2014; Costa, Lambert, Borba, & de Queiroz, 2007; Silva‐Pereira, de Camargo Smidt, & Borba, 2007). For example, low F_1_ germination rates and F_1_ pollen sterility, as components of postzygotic isolation, were crucial for reproductive isolation between *Mussaenda pubescens* var. *alba* and *M. shikokiana* (Rubiaceae; Chen et al., 2014). Furthermore, many plants use a broad range of generalist pollinators. Consequently, the roles of pollinator behavior and pollinator diversity as prepollination barriers may be limited especially during incipient speciation or as a reinforcement following secondary contact (Johnson & Steiner, 2000; Kephart & Theiss, 2004; Waser, Chittka, Price, Williams, & Ollerton, 1996).

The family Orchidaceae shows one of the highest rates of speciation within the angiosperms (Tremblay, Ackerman, Zimmerman, & Calvo, 2005). The evolution of pollination systems and their exploitation of pollinator guilds appear to be one of a suite of important factors driving diversification in this lineage (Givnish et al., 2015; Schiestl & Schlüter, 2009; Tremblay et al., 2005). Some authorities suggest that postpollination barriers are less important in the Orchidaceae (Bernhardt & Edens‐Meier, 2014; Dressler, 1993; Xu et al., 2011). However, the prominent role given to native pollinators to maintain interspecific isolation and promote orchid diversification has focused, in large part, on species with deceptive flowers (Dressler, 1993; Schiestl & Schlüter, 2009; Tremblay et al., 2005; Xu et al., 2011). Based on reproductive isolation mechanisms in nonrewarding orchid species distributed through the Mediterranean, Cozzolino and Scopece (2008) proposed that sexually deceptive species, each with a relatively narrow range of potential pollinators, were more likely to rely on prepollination isolation. In contrast, postpollination barriers were few and weak. The opposite arrangement was hypothesized for generalist food‐deceptive species as they were more likely to share the same insect species in local pollinator guilds (Edens‐Meier, Arduser, Camilo, & Bernhardt, 2018). Indeed, postpollination barriers such as pollen–pistil interactions and low hybrid fertility were stronger in the food‐deceptive orchids compared to sexually deceptive species (Cozzolino & Scopece, 2008; Edens‐Meier, Westhus, & Bernhardt, 2013; Scopece, Croce, Lexer, & Cozzolino, 2013; Scopece, Musacchio, Widmer, & Cozzolino, 2007; Scopece, Schiestl, & Cozzolino, 2015; Scopece, Widmer, & Cozzolino, 2008; Xu et al., 2011).

It is estimated, though, that up to two‐thirds of species in the Orchidaceae offer nectar or volatiles as rewards (Cozzolino & Widmer, 2005) but research on interspecific isolation between congenerics offering rewards are less frequent (Nilsson, 1983; Pauw, 2006; Singer & Cocucci, 1997). In two nectar‐secreting genera, *Platanthera* and *Habenaria*, pollinators in the Order Lepidoptera are shared between congenerics. Instead, variation in column architecture between coblooming species, in these genera, results in the placement of pollinaria on different sites on the same pollinators’ bodies. This establishes a prepollination, mechanical mode of interspecific isolation (Nilsson, 1983; Singer & Cocucci, 1997). A similar example was described in the oil‐secreting genus, *Pterygodium* pollinated exclusively by bees in the genus *Rediviva* (Pauw, 2006). This suggests that the hypothesis of Scopece et al. (2008) in food‐deceptive orchids may also apply to some nectar‐rewarding lineages where different orchid populations should share the same pollinators. In particular, this should apply to nectar‐secreting and bee‐pollinated species as most bee species known to pollinate‐rewarding orchids are generalist foragers.

The genus *Spiranthes* Rich. (Orchidaceae, Orchidoideae, Cranichideae, Spiranthinae; sensu Balogh, 1982 and Salazar & Jost, 2012) should offer taxa and populations useful to studies on prepollination vs. postpollination barriers in rewarding orchids as their flowers are known to secrete nectar (Darwin, 1877; Luer, 1975). In particular, *S. sinensis* remains one of the most widely distributed taxa throughout temperate and montane regions of eastern Asia. It is found from the Himalayas north to Siberia at elevations between 200 and 3,200 m (Chen, Gale, & Cribb, 2009) and up to 3,450 m in Lijiang county (Yunnan, China). However, not all authorities regard it as a single species. Dueck, Aygoren, and Cameron (2014) interpreted it as a species complex distributed as far south as New Zealand and Australia where only pink flowering forms are found. Coleman (1933) investigated the floral biology of populations in southern Australia (Victoria) and found it was pollinated primarily by small native bees although the introduced commercial honeybee (*Apis mellifera*) also withdrew pollinaria. Bernhardt (unpublished data) observed and caught honeybees foraging on the pink flowers in New South Wales in 1992 and 2016 and also found they carried the pollinaria of the orchid.

In contrast, within the Himalayas, the *S. sinensis* complex is represented by two, often sympatric, pink and white color forms. Mehra and Kashyap (1986) proposed segregating *S. sinensis* into two species based on karyotype, floral color, and distribution at different elevations. They classified specimens with pink flowers at 1,800–2,300 m as *S. australis,* while those with white flowers at 300–1500 m were *S. sinensis* ss. Surveswaran, Kumar, and Sun (2017) described the white color form from low elevations as a new species. In Lijiang county, though, both white and pink forms are often sympatric and coblooming (Chen et al., 2009). To date, the pollination ecology of the species in China has not been studied.

We observed and quantified a series of isolating barriers between these two, color forms using field and laboratory techniques addressing the following questions. (i) What is the phylogenetic relationship between white and pink forms among other *Spiranthes* species and members of the *S. sinensis* complex outside the Himalayas? (ii) Do these two forms share the same pollinators? (iii) Do these plants express characters that can reduce the frequency of crosses between forms and are some characters more restrictive than others? (iv) If such characters do exist, are prepollination and postpollination barriers of equal importance?

## 2 MATERIALS AND METHODS

### 2.1 Study site and species

We conducted field experiments at Yulong Mountain (27°00′N, 100°10′E), Lijiang, northwestern Yunnan, China based on nine study sites from 2014 to 2016 (Table S1). We found both forms together in four sites. There was no correlation between the distributions of the two color forms and site elevation (see Bernhardt, Edens‐Meier, Grimm, Ren, & Towle, 2017).

**Figure 1:**
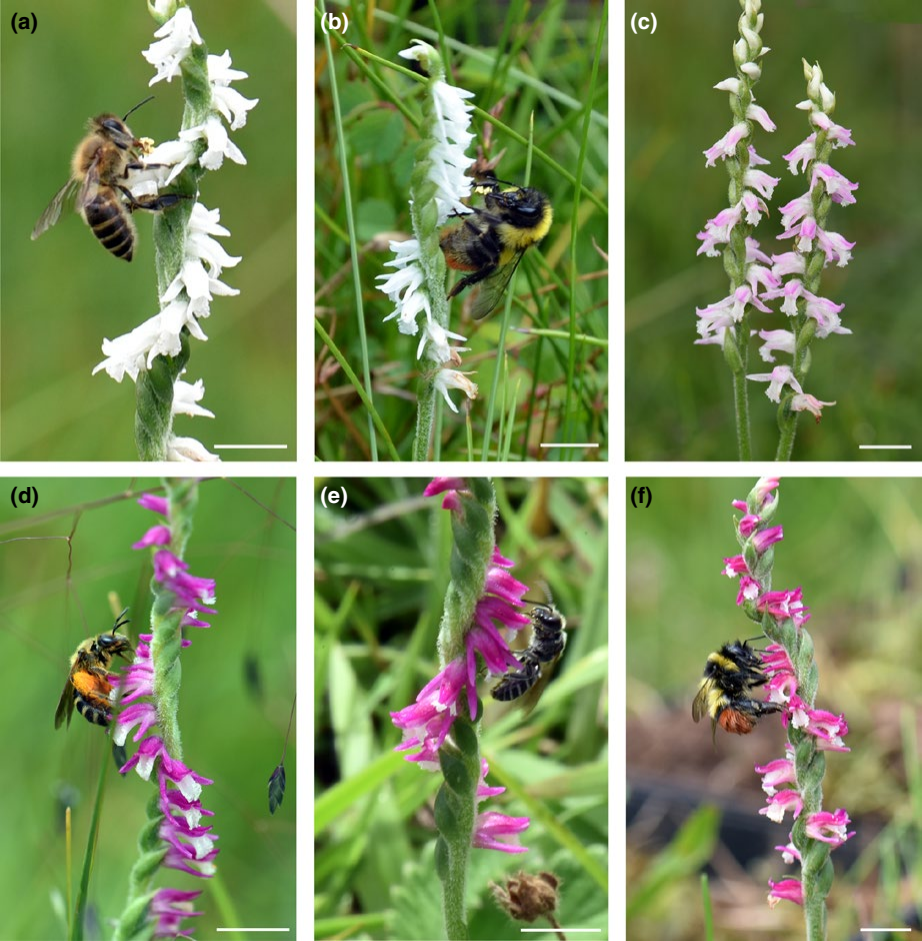
The three color forms (white, pink, and intermediate) of *Spiranthes sinensis* and their insect visitors at a sympatric site SK. (a) *Apis cerana* visiting flowers of the white form (note whitish‐yellow pollinaria on its proboscis). (b) *Bombus friseanus* visiting flowers of the white form (note pollinaria on its proboscis). (c) Intermediate inflorescences in full flower. (d) A female of *Lasioglossum subopocum* (note pollen load on scopa) visiting flowers of a pink form (note white labellum). (e) A female of a *Lasioglossum* sp. visiting flowers of a pink form. (f), *B. friseanus* foraging for nectar on flowers of a pink form

Flowers of *S. sinensis* sl are tubular‐campanulate as in most spiranthoid orchids sensu Salazar, Chase, Arenas, and Ingrouille (2003) and Salazar and Jost (2012). Based on herbarium records (i.e., PE, KUN from 1910 to 2012) and our observations, white morphs of *S. sinensis* are found in many sites throughout China. Both color forms have a white or whitish labellum but the white form also has white sepals and lateral petals (Figure 1a,b). The same organs are dark pink‐magenta in the second form (Figure 1d–f). A third, intermediate form, in which white sepals and lateral petals blush a light or dusky pink at their apices (Figure 1c) was found in some sites but occurred in insufficient numbers for most of the tests and experiments (Table 1). In Lijiang, white and pink forms often show sympatric distributions, but white forms occur in greater numbers in wet, humid meadows. Pink forms are more common on the upper, drier slopes of grasslands (Table 1 and Figure 1). Except for floral color and scent, four other floral morphological traits flower length (from the terminus of the tube opening made by the curving labellum and dorsal sepal down to the bases of perianth segments), flower tube opening length (the distance between the labellum and the dorsal sepal), flower opening width (the distance of the tube opening between the two lateral petals), and labellum width showed no differentiation among the three color morphs by principal coordinate analysis (PCoA, Figure S1). Nectar secretions were observed at the bases of the labella produced by two, basal, globose callosities. Nectar samples were too minute to analyze further.

**Table 1:** Comparative ecology, floral presentation, and cytology of the three forms of *Spiranthes sinensis* in Lijiang (Yunnan)

Color morph	White form	Pink form	Intermediate form
Habitat	Wet grasslands, marshes, banks of rice paddies	Upper dry grasslands, among bushes, forest edges, wasteland	Dry grasslands, in the bushes, forest edge, rice paddy banks
Density (No. individual/m^2^)	ca. 2–25	ca. 1–10 (rarely 15)	ca. 0.1–1
Flowering phenology	July–September	July–October	August–September
Flower color	White	Pink	White with light pink sepal and lateral petal tips
Flower odor	Aromatic	Odorless	Aromatic
Karyology	2*n = *30	2*n = *30	2*n = *30

### 2.2 Base chromosome number and phylogenetic analysis

To count chromosome numbers in the three color forms, we cut off actively growing root tips from living transplanted specimens (see below). These root tips were pretreated with 0.1% colchicines then fixed in Carnoy I (3:1, 95% ethanol: glacial acetic acid) at room temperature for 4 hr. Fixed root tips were macerated in 1 mol/L hydrochloric acid at 60°C for 10 min, stained in carbolfuchsin for 4 hr and then squashed in a drop of 45% formic acid on microscope slides.

Leaf tissues were collected in the field and dried with silica gel. Genomic DNA was extracted using a modified CTAB method (Doyle & Doyle, 1987). Two chloroplast genes (*matK* and *trnS‐G*) were sequenced. Protocols for PCR amplification and sequencing follow Yu et al. (2011). All new raw sequences were assembled and edited using SeqMan software (DNAstar packages, Inc.). Preliminary alignments were produced using Muscle version 3.8.31 (Edgar, 2004). Sequence data matrices were concatenated using Sequence Matrix version 1.7 (Vaidya, Lohman, & Meier, 2011). To compare phylogenetic relationships, we generated trees based on *trnS‐G* and *matK* for our three color forms of *S. sinensis* (including three samples of field‐collected *S. australis* from NSW, Australia). Specimens used and relevant information are listed as supporting information (Table S2). Two additional individuals of *S. sinensis* were used previously in the phylogeny generated by Dueck et al. (2014) and downloaded from Genbank. We also downloaded *trnS‐G* and *matK* sequences of *S. aestivalis* (two individuals), *S. spiralis* (one individual), and *S. tuberosa* (five individuals) from Genbank. Maximum likelihood analyses were implemented in MEGA (version 5.05), with GTR+G model and 1000 nonparametric bootstrapping replicates. Voucher and GenBank accession numbers for samples are listed in Table S2.

### 2.3 Phenological Isolation

To quantify the degree of overlap of the flowering periods of both forms, we selected one 20 × 1.5 m^2^ site where white (*n = *77) and pink forms (*n = *40) were sympatric and all individuals were tagged. We recorded the day on which the first flower on each scape opened, and expansion of the corolla tube was visible, until the wilting of the tube on the last open flower on the same scape from July to September 2016. We visited the plot every 4–6 days. As the average life span of a flower was similar in both color forms (about 10 days), we assumed that all flowers had the same opportunity to participate in pollination events. We then calculated the strength of phenological asynchrony as a barrier following Sobel and Chen (2014):

(1) RI=1−(S/(S+U))

where *S* refers to the proportion of flowering time that is shared between the two forms, and *U* refers to the proportion of unshared flowering time.

### 2.4 Floral reflectance and bee vision

We measured color reflection of the abaxial surfaces of the dorsal sepal and lateral petals of flowers (*n = *35 for white form; 31 pink form and 10 intermediates) in sympatric sites using an USB2000+ spectrometer with an PX‐2 pulsed xenon light source (Ocean Optics, Dunedin, FL, USA). All measurements were made within the 300–700 nm range with increments of 0.37 nm.

We used the color hexagon model to calculate the chromatic contrasts among the dorsal sepals of white, intermediate and pink color forms with the honeybee and bumblebee subjective model (Chittka, 1992) to evaluate whether each flower form can be discriminated by these apids. In our consideration of the conservatism of color vision in the genera *Apis* and *Bombus*, we used the standard photoreceptor sensitivities of *A. mellifera* L. (Chittka, 1992) and *B. lapidarius* L. (Peitsch et al., 1992) for honeybees and bumblebees. We fixed a minimum threshold of 0.01 known to be just noticeable differences (JNDs) for color discrimination by both *Apis* and *Bombus* species according to Chittka, Gumbert, and Kunze (1997). We conducted all analyses in R software by using the pavo package (Maia, Eliason, Bitton, Doucet, & Shawkey, 2013).

### 2.5 Floral volatile analysis

The collection of floral scents followed a dynamic headspace collection method as described by Edens‐Meier, Raguso, Westhus, and Bernhardt (2014). Upon completion of collection, scent traps were eluted into a 1.5 ml borosilicate glass autosampler vials using 300 μl of GC‐MS grade hexane and stored at −20°C. In total, we sampled eight white flower inflorescences with each scape bearing 24–32 open flowers. We sampled four pink inflorescences each bearing 19–29 flowers. The sampling period for each inflorescence was three hours at ambient temperatures of 20.1°C. Floral headspace samples eluted in hexane were concentrated to 50 μl under a flow of nitrogen gas (N2). An internal standard of 5 μl of a 0.03% solution of toluene in hexane was added to each sample. The volatiles were analyzed on a Hewlett Packard Hp 6890 Series GC System coupled to a Hewlett Packard 5973 Mass Selective Detector. An Hp‐5MS column (5% Phenyl‐methylpolysiloxane; 30 m long; inner diameter 0.25 mm; film thickness 0.25 μm; Agilent, USA) was used for analyses. Each one‐μl sample was injected at 240°C. Electronic flow control was used to maintain a constant Helium gas flow of 1.0 ml/min. The GC oven temperature began at 40°C and was increased at 3°C per min to 80°C, then increased 5°C per min to 280°C, and held for 20 minutes. The MS interface was 250°C, and the ion trap worked at 230°C. The mass spectra were taken at 70 eV (in EI mode) with a scanning speed of one per scan from m/z 35 to 500. Component identification was carried out using NIST 05 mass spectral database and Wiley 7n.1.

### 2.6 Pollinator observation and collection

Insect activity on flowers was observed on sunny days from 09:30 to 16:30 from July to September for three consecutive years (2014–2016) over a grand total of 89 days, *n = *168 hours for the white form and *n = *262 hr for the pink form. More hours were spent observing the pink form because it received more visits from small‐bodied insects that were difficult to see and catch. In addition, we observed floral foragers at night from 19:30 to 22:00 and from 00:00 to 06:30 by using a red‐light torch at YSZ in August 2016. We completed a total of 50 and 35 nocturnal, observation hours, respectively, for white and pink forms. We restricted collections of insects to those observed landing on inflorescences and then ascending the scape while probing flowers. These specimens were netted and euthanized in jars with fumes of ethyl acetate following Edens‐Meier and Bernhardt (2014). Specimens were pinned, labeled, measured, and sent to entomologists for identification. Measurements included length, width, and thorax depth following Ren, Wang, Bernhardt, Camilo, and Li (2014). Vouchers were deposited at the Kunming Institute of Botany, Chinese Academy of Sciences (CAS), Kunming.

### 2.7 Pollinator fidelity/isolation (controlled experiment)

We dug up 20 white and 20 pink flowered plants, while all flowering stems were in bud and replanted each on in its own plastic pot. We covered each inflorescence with a muslin bag, tied at its base, to prevent insect visitation. On sunny days, we moved potted plants to do the following controlled choice experiments. For each experiment, 20 × 20 color pairs (white and pink) were tested with a pot of one form placed in an alternating pattern with the second form. The spatial separation treatments were performed with the distance of 25 cm between the potted morphs. This distance approximated the closest distance between two color forms as observed in sympatric sites. To avoid the influence of context choice experience by insects, we chose a site that was isolated from all other sites where known forms of *S. sinensis* grew at least 100 m away. This site was further isolated from resident populations by a native *Pinus*–*Quercus* forest with an interlocking canopy where *S. sinensis* did not grow. We recorded these observations for 28 days. We recorded the species of floral foragers, the number of flowers they visited, the number of color forms each pollinator visited in each foraging bout, and the sequence of individual foraging bouts. This included interform visitations. After this experimental series finished, we transferred the potted individuals to the Lijiang field station glasshouse to protect them from poachers (Bernhardt et al., 2017). Each procedure lasted approximately 3 to 5 hr.

The pollinator foraging preferences for each color form was calculated following Sobel and Chen (2014) and Brys, Cauwenberghe, and Jacquemyn (2016), using the equation:

(2) RI=1−2×(H/(H+C))

where *C* refers to the proportion of intraform pollinators foraging, and *H* refers to the proportion of interform pollinators foraging between white and pink form of *S. sinensis*. Based on this experiment of foraging choices, Gegear's constancy index (CI) was used to calculate the constancy of individual pollinators (Gegear & Thomson, 2004):

(3) CI=(c−e)/[(c+e)−2ce]

where “*c*” is the actual proportion of transitions between the same color forms, and “*e*” was the expected proportion of transitions between the same color form based on the overall frequency of one specific color form. If *p* is the proportion of visits to one specific color form, then *e *= *p* ^2^ + (1 − *p*)^2^. Possible values range from −1 (complete inconstancy) to 0 (random foraging) to 1 (complete constancy).

### 2.8 Pollinia–pistil interactions

Selected inflorescences at our field sites were bagged in bud (see above). As flowers opened, they were subdivided into three‐hand pollination treatments. (i) Self‐pollination in which the pollinarium was removed and deposited on the stigma of the same flower. (ii) Intraform cross‐pollination in which a pollinarium was removed from one flower and then deposited on the stigma of a flower on a second inflorescence growing at least 10 m away. (iii) Interform pollination in which a pollinarium was removed from flowers of both forms and then deposited on the stigma of the other color form that had its pollinarium removed. (iv) Controls were never hand pollinated and reflected natural rates of mechanical self‐pollination (autogamy). Therefore, in all hand‐manipulated experiments, a stigma received the entire pollinium of one anther from one flower regardless of cross.

Pistils of hand‐pollinated flowers were harvested seven days and 14 days later. Pistils were excised, fixed in 3:1 95% ethanol:glacial acetic acid, and preserved in 70% ethanol prior to softening. Softened pistils were placed on glass slide and stained with decolorized aniline blue, and tissues were spread under a coverslip (Edens‐Meier et al., 2010). Each specimen was observed under an epifluorescence microscope (Axio Lab.A1, Zeiss, Oberkochen, Germany). However, as pollen tube germination/penetration in orchids produces hundreds to thousands of tubes in one pistil and it is not possible to count the number of tubes/pistil until tubes penetrate gynoecium tissue (Edens‐Meier et al., 2010, 2013). Therefore, we recorded the number of pollen tubes entering the ovary for each pollination treatment of each color form. RI of pollinia–pistil interaction was calculated using Equation (2) following Sobel and Chen (2014) where *C* refers to number of intraform pollen tubes entered the ovary; *H* refers to number of interform pollen tubes entered the ovary.

### 2.9 Fruit production

We did parallel hand pollination experiments following the same treatments as above in 2015 and 2016 but these treated flowers were allowed to develop into capsules. At the end of September, fruit production was recorded by counting and collecting capsules of hand‐pollinated flowers. Capsules were collected for further seed development experiments (see below). Equation (2) was again used to calculate the RI of fruit production following Sobel and Chen (2014), where C refers to fruit sets from intraform pollination, and H refers to fruit sets from interform pollination.

### 2.10 Seed development

All the seeds in each capsule were extracted and emptied onto separate Petri dishes. Seed development was checked under an Olympus BX51 microscope (Tokyo, Japan) using the methods of Ren et al. (2014) by scoring about 300 seeds in each fruit. Seeds were categorized as containing either large, or small (half sized), or aborted or empty (no embryo; see Ren et al., 2014). We used the rate of large embryos to evaluate seed development. We used Equation (2) to calculate RI of seed development following Sobel and Chen (2014), where *C* refers to large embryo rate from intraform pollination, and *H* refers to large embryo rate from interform pollination.

Fruits produced by natural (insect‐mediated) pollination were harvested at five sites for the white form and three sites for the pink form in 2016, see Supporting Information, Table S1. We also observed rates of natural fruit set on 11 flowering stems of the intermediate plants at site SK in 2016. Ripe capsules were collected at YLSK and SKD (*n = *17). Their embryos were scored as above.

### 2.11 Estimating total isolation and relative contributions of barrier strengths

Total RI between white and pink form of *S. sinensis* was calculated as follows:

(4) $${\text{RI}}_{\mathrm{total}}=1-2\times \frac{S\times H_{S}+U\times H_{u}}{\left(S\times H_{S}+U\times H_{u}\right)+\left(S\times C_{S}+U\times C_{u}\right)}$$

where S refers to the extent of the shared period of flowering, and *U* refers to the unshared period of flowering. *H* and *C* represent heterospecific (interform in the study) and conspecific effects (interform in the study), respectively, but are multiplied across all components of RI and are both considered within the shared (*H*_*S*_, *C*_*S*_) and the unshared (*H*_*U*_, *C*_*U*_) period of flowering (see Sobel & Chen, 2014). To calculate the absolute contribution (AC_*i*_) of each of the studied barriers to total isolation, the individual strength of a barrier was discounted by the impact of previously acting barriers as follows:

(5) $${\text{AC}}_{i}={\text{RI}}_{\left[1,i\right]}-{\text{RI}}_{\left[1,i-1\right]}$$

The relative contribution (RC_*i*_) of each barrier to total isolation was calculated using the general equation from Ramsey, Bradshaw and Schemske (2003):

(6) $${\text{RC}}_{i}={\text{AC}}_{i}/{\text{RI}}_{\mathrm{total}}$$

We calculated the asymmetry of each barrier as the absolute value of the difference between the strengths of a given barrier for both crossing directions (white form as pollen donor and pink form as pollen recipient vs. pink form as pollen donor and white form as pollen recipient) following Lowry, Modliszewski et al. (2008) and Lowry, Rockwood et al. (2008).

### 2.12 Statistical analyses

All analyses were conducted using R computational environment (R Development Core Team, 2016). We compared the color hexagon distances between color morphs through a pairwise comparison, against the minimum discrimination threshold by performing a one‐sample t test. We used the Kruskal–Wallis nonparametric analysis of variance for each color morph to compare the physical measurements of pollinators, floral constancy, the number of pollen tubes penetrating each ovary, fruit set, and the ratios of large embryo. We also used a Dunn's post hoc test (Dinno, 2016) to determine pairwise differences for the former analyses.

Differences in fruit production between pollination treatments were assessed using two Generalized Linear Models (GLMs) with binomial errors distribution and a logit link function for pink and white forms, respectively. Pollination was treated as a fixed effect and fruit production (“set no fruit” coded as “0” and “set fruit” as “1”) as a binary response variable. For the ratio of large embryos (see above) to the three remaining categories, we first transformed ratio data using an arcsine transformation to meet the assumptions of the test. Comparisons of ratios of large embryos among pollination treatments were assessed using GLM with Gaussian errors and an identity‐link function. Pollination treatment was assessed as a fixed effect and transformed ratio data as a response variable. Then, we assessed significances of all GLM models mentioned above with likelihood‐ratio tests using the ANOVA function in R package car (Fox & Weisberg, 2011). Post hoc multiple comparison tests using the glht function in multcomp package (Hothorn, Bretz, & Westfall, 2008) were used to detect for differences between pollination treatments.

## 3 RESULTS

### 3.1 Molecular phylogeny and chromosome number analysis

**Figure 2:**
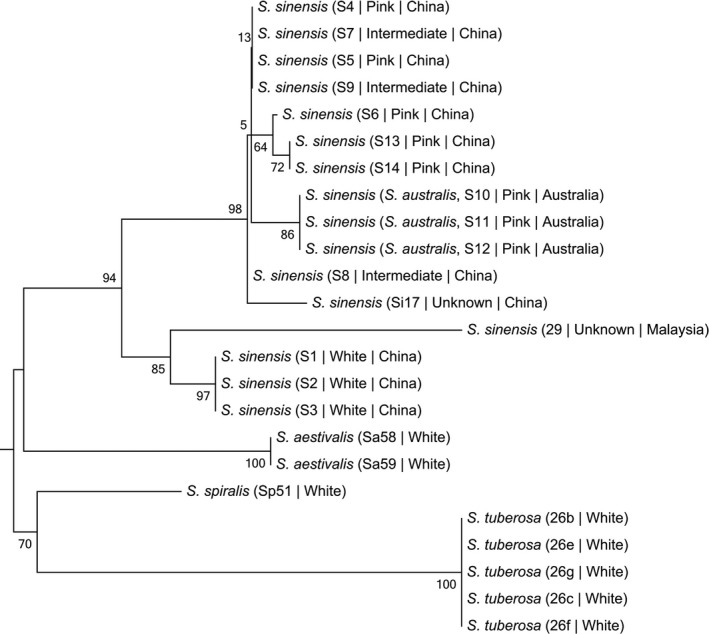
ML tree of the three forms of *S. sinensis* sl and three related *Spiranthes* spp.: *S. aestivalis*,* S. spiralis*, and *S. tuberosa* reconstructed from *trn*S‐G and *mat*K. Numbers at each node are bootstrap support values. S1–S3: white color form, S4–S6, S13–S14: pink color form, S10–S12: *S. sinensis* (*S. australis*), pink form, S7–S9 intermediate form

Both white and pink color forms of *S. sinensis* are diploid with the same chromosome numbers (2*n = *30). *Spiranthes sinensis* sl is a monophyletic clade (ML, 91%). Pink and intermediate (dusky‐blush petals, see above) forms showed a well‐defined clade (ML, 99%) segregating from the white form. Pink forms of *S. sinensis* sl in China are more closely allied to pink *S. sinensis* in Australia then either is to the white form in China (Figure 2). Regardless of color form, all samples of *S. sinensis* sl were more closely related to each other than to any of the remaining three species (Figure 2).

### 3.2 Phenological isolation

At site SK, the white form flowered from the 24th July to 9th September, while the pink form flowered from 31st July to 14th September. Therefore, the flowering phenology of these two, color forms overlapped broadly. RI_phenology_ for the white form as female parent is 0.15. The RI_phenology_ for the pink form as female parent is 0.11. The value of asymmetry in this barrier was 0.04.

### 3.3 Floral isolation based on floral reflectance

**Figure 3:**
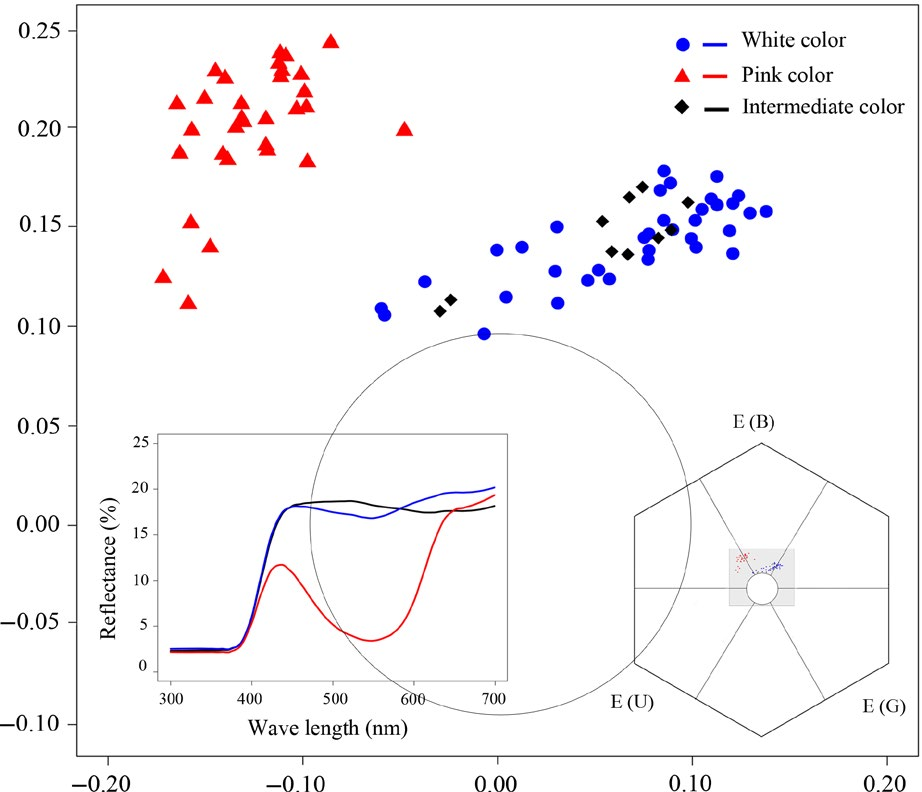
Color loci of stimuli in the color hexagon of honeybee vision. The color space inside the central circle (<0.1 hexagon units) appears achromatic to the bees. The gray fraction in the color hexagon was amplified. Blue circles represent the loci of sepals in the white forms, red triangles represent the loci of dorsal sepals in the pink forms, and diamonds represent the loci of sepals in intermediate forms in the color hexagon

The spectrum reflectance curves of the pink form were different from the intermediate and white forms (Figure 3). The color distance between pink and white forms (0.21 ± 0.06 hexagon units; mean ± *SD*;* t* = 26.61, *p *<* *.001) and between pink and intermediate forms (0.19 ± 0.05 hexagon units; *t* = 9.41, *p *<* *.001) were significantly higher than the discrimination criteria (Just noticeable differences, 0.1 hexagon units). The distance between the white and intermediate color form (0.06 ± 0.02 hexagon units) was significantly lower than the discrimination criteria (*t* = −5.36, *p *<* *.001). The *Apis mellifera* and *Bombus lapidarius* vision models showed similar results (Figure S2).

### 3.4 Volatile molecules in floral scent

After excluding the contaminated molecules in the Control, we failed to detect any flower‐related volatiles in the pink forms. In white forms, a single dominant floral compound, 2‐Phenylethanol, was detected with an emission rate of 571.45 ± 180.60 ng inflorescence^−1^ hr^−1^ (*n = *8). Traces of other scent molecules associated with orchid flowers were never detected.

### 3.5 Pollinator observation

No floral foragers were observed visiting the flowers of these two color forms at night. During daytime, a total of 83 flower‐visiting insects, representing two genera in the family Apidae (*Apis cerana*,* Bombus friseanus* and *B. grahami*), were observed visiting the white form with 75% of the collected specimens carrying 1–6 pollinaria on their proboscides (Figure 1a,b). Small, solitary bees were never observed on the white form. A total of 50 insects, representing 12 species in the families Apidae (four species in the genera *Apis*,* Bombus* and *Ceratina*), Halictidae (six species in the genera *Halictus* and *Lasioglossum*), and Megachilidae (two species in the genera *Anthidium* and *Hoplitis*) foraged on the pink form (Figure 1d,e). Visits from *A. cerana* and *B. friseanus* to the pink form were infrequent (Figure 1f). *Ceratina flavipes* (Apidae) was the most commonly observed species on the pink form accounting for 42% of total visits. These bees carried 1–3 pollinaria on their proboscides. At sites where only the white form bloomed, *Ceratina* and the other small‐bodied, solitary bees (see above) were observed but these insects confined their visits to the flowers of coblooming *Parnassia wightiana* and *Potentilla lancinata*. Conversely, at sites where only the pink form bloomed, we noted that *Apis cerana* and the resident *Bombus* species were more likely to visit flowers of coblooming *Pedicularis cephalantha*,* Prunella hispida,* and *Ligularia vellerea*. We found no site‐specific patterns in pollinator visitation. We observed that *B. friseanus* visited both white and pink forms in sympatric populations at DZW and YSZ in 2016. On one occasion at site YSZ (2016), we observed one *B. friseanus* visiting a white form before switching to a pink form, and then we observed another *B. friseanus* visiting a white form before switching to a pink form. *Apis* and *Bombus* species visiting the white form were significantly larger than solitary bees visiting the pink form in body length (Kruskal–Wallis test, χ^2 ^= 44.78, *df* = 1, *p *<* *.001), thorax width (χ^2* *^= 29.54, *df* = 1, *p *<* *.001), and thorax depth (χ^2 ^= 29.44, *df* = 1, *p *<* *.001).

### 3.6 Pollinator‐mediated isolation

We recorded 56 bees visiting 2–27 inflorescences (9.92 ± 7.56) during foraging bouts in the plot containing potted plants of both color forms. This resulted in a total of 474 transitions among inflorescences, with 434 by visits of bees to the same color morph (91.56% of total transference, 370 White→White, and 64 Pink→Pink) and 40 visits between the two color forms (8.44%, 20 White→Pink and 20 Pink→White). *Bombus friseanus* was responsible for the majority of interform visits (see above). Only one *A. cerana* was observed to visit a white and a pink form. The flower constancy index for *A. cerana* (0.94 ± 0.33, *n = *30) was significantly higher than for *B. friseanus* (0.82 ± 0.31, *n = *26; Kruskal test, χ^2^ = 5.46, *df* = 1, *p *<* *.05; Table 2). Based on this controlled series, the strength of reproductive isolation due to selective foraging by pollinators for white and pink forms as female parents were 0.88 and 0.52, respectively. The value of asymmetry in this barrier was 0.36.

**Table 2:** Intraform vs. interform foraging bouts by bees. Gegear's constancy index (CI, mean ± *SD*) is shown for white and pink color forms (w vs. p)

Pollinators	Interform/total bouts	No. of floral visits				CI
		w→w	p→p	w→p	p→w	
*Apis cerana*	1/30	237	–	1	1	0.94 ± 0.33
*Bombus friseanus*	7/26	133	64	19	19	0.82 ± 0.31

Dash “–” indicates a solitary case where no pollinator visits were observed.

### 3.7 Pollinia**–pistil interactions**

When a whole pollinium was placed on a stigma, it germinated and penetrated style tissue (>100 pollen tubes) entering the ovary after 7 days regardless of color morph. The difference between the total number of pollen tubes penetrating ovaries between seven and 14 days following hand pollination was not significant (χ^2^ **= 0.01, *df* = 1, *p *=* *.92). The total number of pollen tubes penetrating ovaries in self‐ vs. intraform, vs. interform pollination treatments using the white form as the female parent was 146.30 ± 120.23 (*n = *30), 118.93 ± 99.52 (*n = *30), and 125.62 ± 115.24 (*n = *29), respectively, and when the pink form was the female parent, pollination treatments were 42.33 ± 43.43 (*n = *18), 83.95 ± 60.81 (*n = *19), and 69.75 ± 80.54 (*n = *20). The difference in the number of pollen tubes penetrating ovaries at 14 days following pollination among self‐form, intraform, and interform flowers was not significant for both white (χ^2 ^= 0.88, *df* = 2, *p *=* *.64) and pink ovaries (χ^2^ = 3.44, *df* = 2, *p *=* *.18). The RI of this barrier was low for white and pink forms as female parents were at −0.03 and 0.09, respectively. The value of asymmetry in this barrier was 0.12.

### 3.8 Fruit production

**Figure 4:**
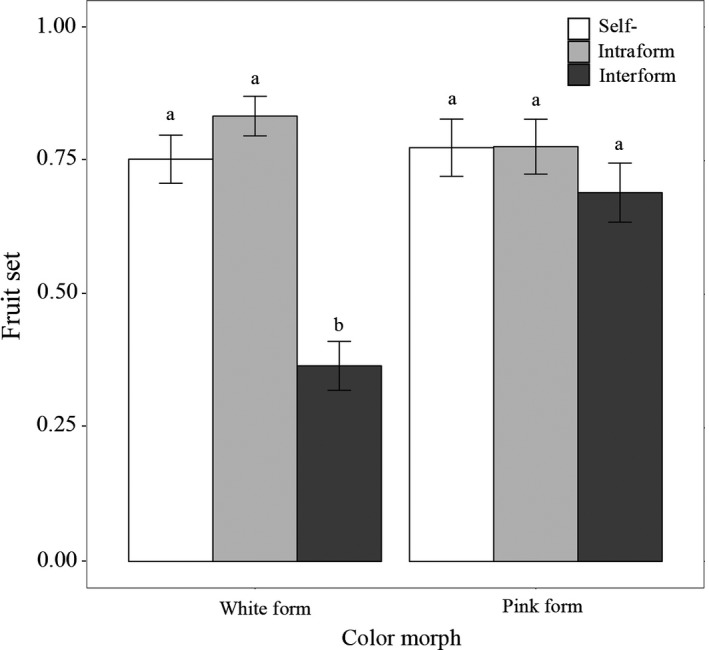
Fruit set following self‐, intraform and interform pollinations for white and pink forms used as female parents (ovule donors). Different lower case letters indicate significant differences (*p *<* *.001)

Control (mechanically autogamous, self‐pollinated flowers) never set fruit in either color form. The number of capsules produced showed significant differences among pollination treatments (χ^2 ^= 47.24, *df* = 2, *p *<* *.001) and between color forms (χ^2 ^= 6.43, *df* = 1, *p *<* *.05). In particular, for pollination treatments among white forms, the number of capsules produced by the interform cross was significantly lower than results for self‐pollination (*p *<* *.001) and intraform pollination (*p *<* *.001). However, when the pink color form was used as a female parent, there were no significant differences among the number of capsules produced in the three pollination treatments (χ^2^ = 1.71, *df* = 2, *p *=* *.42; Figure 4). Interform isolation in the white and pink forms as female parents for fruit production were 0.39 and 0.06, respectively. The asymmetric value in this barrier was 0.33.

Natural rates of fruit set in the 11 infructescences of intermediate forms from YLSK was 0.35 ± 0.20. This was significantly lower than the surrounding white (0.80 ± 0.17, *n = *280, sites = 5; χ^2^ = 27.49, *df* = 1, *p *<* *.001) and pink infructescences (0.76 ± 0.25, *n = *159, sites = 3; χ^2 ^= 21.63, *df* = 1, *p *<* *.001).

### 3.9 Seed development

**Figure 5:**
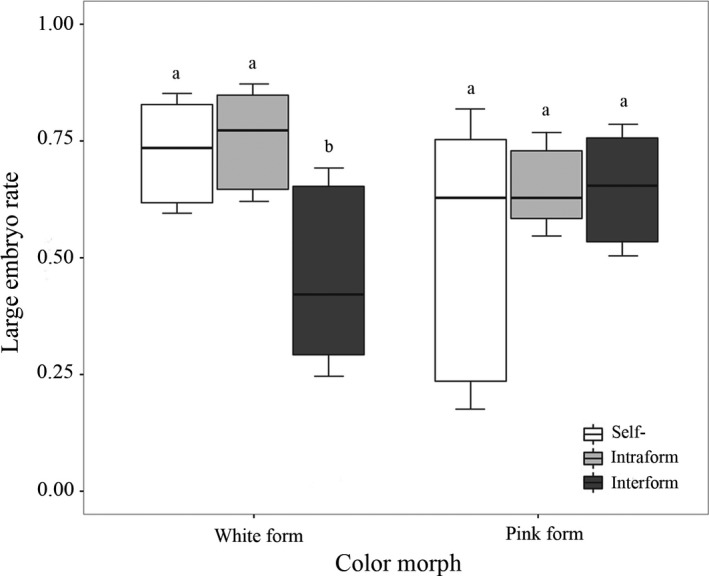
Comparison of embryonic development in seeds among self‐, intraform and interform pollinations for white and pink as female parents. Different lower case letters indicate significant differences (*p *<* *.001)

Significant differences in the proportion of seeds with large embryos were found among the three pollination treatments when the white form was a female parent (χ^2* *^= 34.18, *df* = 2, *p *<* *.001; Figure 5). Less than half of the interform‐pollinated seeds had large embryos (45.51 ± 23.20%; *n = *33). This was significantly lower than the proportion of large embryos in both the self‐pollinated fruits (71.94 ± 13.33%, *n = *33; *p *<* *.001) and intraform‐pollinated seeds (72.42 ± 16.08%, *n = *33; *p *<* *.001). When pink forms were used as female parents, there were no statistical differences among the rates of large embryo development in self‐pollination (50.85 ± 29.45%, *n = *22), intraform pollination (65.48 ± 13.46%, *n = *29), and interform pollination (63.35 ± 20.94%, *n = *29; χ^2 ^= 4.21, *df* = 2, *p *=* *.12) categories. Reproductive isolation via seed development for the white and pink form as female parents was 0.23 and 0.02, respectively. The value of asymmetry in this barrier was 0.21.

Production of large embryos by intermediates was also low pooling seeds from YLSK and SKD sites (15.34 ± 14.53%, *n = *17) compared to naturally pollinated white (79.51 ± 17.60%, *n = *70, sites = 2; χ^2 ^= 37.10, *df* = 1, *p *<* *.001) and pink forms (66.69 ± 21.94%, *n = *28, sites = 1; χ^2 ^= 26.52, *df* = 1, *p *<* *.001).

### 3.10 Total isolation

**Figure 6:**
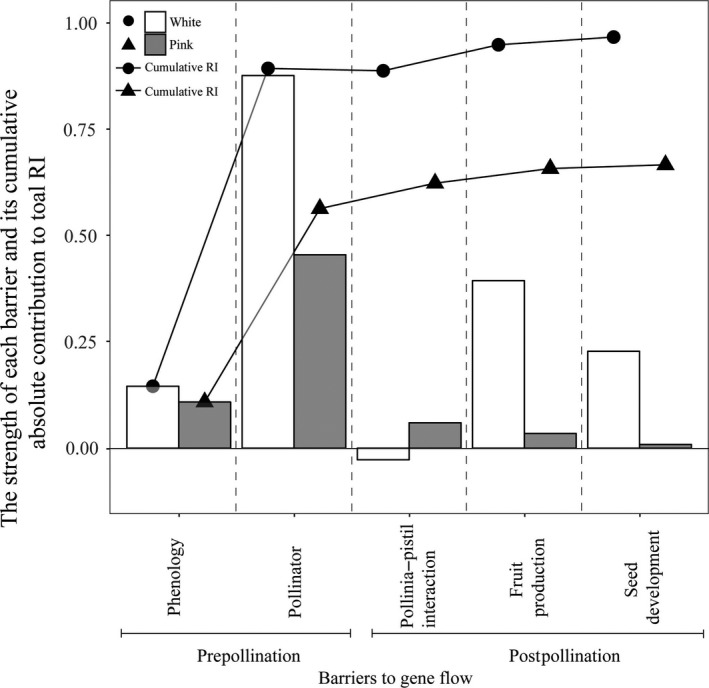
Absolute contributions of five sympatric barriers to total isolation in reciprocal crosses between two color form of *Spiranthes sinensis* sl. The line graph represents the cumulative contribution to RI of a mechanism after accounting for each of the previous mechanisms investigated

The total reproductive isolation was 0.97 for the white and 0.68 for the pink as the female parent when sympatric. The highest contributions were found at the stage of pollinator visitation (see Figure 6 and Table S3) compared to other isolating barriers (e.g., phenology, fruit production and seed developments). The relative contributions of each of these individual barriers to total RI varied between −0.006 and up to 0.77.

## 4 DISCUSSION

### 4.1 Genetic analyses

Our phylogenetic tree showed clearly that *S. sinensis* sl segregates from some other known *Spiranthes* species, and the Himalayan white form segregates from pink forms found in the Himalayas and eastern Australia. However, the pink does not segregate from the rare intermediate. Due to uniparental inheritance, we concur that the chloroplast genome is unsuitable to investigate gene flow in recently diverged lineages, and there are two ways to interpret the genetic evidence provided. First, as the intermediate form is found only where pink and white are sympatric, it may be best interpreted as a recurrent hybrid. The fact that intermediate and pink forms are polyphyletic follows other cases of interspecific hybridization in which some characters in the *F*_1_ are shared with only one parent and do not intergrade (Bernhardt & Edens‐Meier, 2014; Borba et al., 2001; Edens‐Meier et al., 2013; Silva‐Pereira et al., 2007). Our results comparing fruit set and embryo development in the intermediates suggest some degree of hybrid sterility in intermediates but further investigations are required. A second possibility is that the intermediate form merely represents a natural gradation of pigmentation and reproductive success in the pink form that could be associated with younger, but sexually mature plants. This can only be resolved using genetic markers in the nuclear genome to estimate levels of gene flow within sympatric and allopatric populations of this species. In either case, though, it is most unlikely that the intermediate represents a rare, third morph within an unbalanced, floral color polymorphism (sensu Futuyma, 2013 and Bernhardt et al., 2016) as in some *Thelymitra* species (Edens‐Meier & Bernhardt, 2014).

Our pink and white forms contained the same number of chromosomes and these counts paralleled earlier analyses of the same species in other regions of the western Himalayas see Mehra and Kashyap (1986). This was also consistent with an earlier review by (Dressler, 1993) in which 2*n = *30 was regarded as basal for both the genus *Spiranthes* and its allies. There is no evidence of polyploidy in our white form compared to the hybrid origin of white flowered, allotetraploid *S. hongkongensis* (4*n = *60; Sun, 1996). Of course, full karyological analyses of our two forms are still required to see if there are any pronounced differences in chromosome structure.

### 4.2 Comparative roles of prepollination vs. Postpollination barriers

There is little evidence of phenological isolation at our sites with flowering periods overlapping between the two forms when they are sympatric. This is to be anticipated as populations occurred at different elevations. Presumably, isolated populations had different flowering periods based on local differences in climate.

Pollination by polylectic and/or polyphagic bees are common within the Orchidaceae (Dressler, 1993; Nunes et al., 2017; van der Pijl & Dodson, 1969). Darwin (1877) may have been the first to document the pollination of a *Spiranthes* species by a *Bombus* species. Floral presentation (e.g., flowering pattern, color, scent etc.) in our two forms appears to have diverged sufficiently to affect some degree of reproductive isolation even when flowering periods overlap (see above). Our observations and analyses suggest that differences in floral color and scent production may influence the foraging of different pollinators at the same site. Floral color is an important isolation mechanism in pollinator shifts from bees to hummingbirds (Bergamo, Rech, Brito, & Sazima, 2016; Bradshaw & Schemske, 2003; Lunau, Papiorek, Eltz, & Sazima, 2011). Its role in this study is less clear as white and pink perianth segments may affect different responses in honeybee and bumblebee vision models. We must note,though, that Schiestl and Schlüter (2009) concluded that flower color was generally less important for floral isolation in most orchid species compared to parallels between floral and pollinator dimensions. In fact, based exclusively on flower colors and floral measurements, our two forms were poorly isolated as the deposition of pollinaria on foragers was restricted to the bases of the proboscides. This was not comparable to the interspecific and intergeneric isolation described in the pollination of related epidendroid orchids (Dressler, 1993) sharing the same euglossine bee pollinators. These taxa remain isolated by morphological differences in column and labellum architecture (*Pedicularis‐*type isolation, sensu Nunes et al., 2017 and Schiestl & Schlüter, 2009). When the same species of euglossine bee collects scents from several coblooming orchids, pollinaria are deposited on different dorsal and ventral parts of the bee's body (head vs. thorax, vs. abdomen vs. leg) avoiding interspecific hybridization (Dressler, 1981).

Instead, our two forms differed dramatically in scent production. To our knowledge, this is the first study to examine the chemical composition of floral scent in the genus *Spiranthes* or tribe Spiranthinae. Pink forms appeared devoid of detectable levels of volatiles. The white form produced one molecule but it was a most important molecule as 2‐Phenylethanol (2PE) has been identified repeatedly in the flowers of other orchids (Edens‐Meier et al., 2014) and is also associated with at least one fly‐bee‐pollinated basal angiosperm (Bernhardt et al., 2003). The same molecule is shared in lineages belonging to a number of unrelated families (Kaiser, 2010). The absence of this “white scent” (sensu Kaiser, 2010) may mean that pink pigmentation is sufficient to attract halictids and *Ceratina* species but color alone may be insufficient to attract larger *Bombus* and *Apis* species consistently to pink flowers. Floral constancy increases when artificial flowers differ in multiple characteristics such as color, scent, and size (Gegear, 2005). Consequently, another possible function of 2‐phenylethanol in white flowers maybe reinforce the signal of the white flowers and enhance flower constancy for *Bombus* and *Apis* via associative learning. Curiously, when the emission rate of 2PE is high (457.10 ng inflorescence ^−1^ hr^−1^), this molecule is also reported to repel visitation by *Bombus* species (Galen, Kaczorowski, Todd, Geib, & Raguso, 2011). We also note that the dominant presence of *A. cerana* on the white form is also novel as past reviews of the literature do not often associate *Apis* species with orchid pollination (see review in Attri & Kant (2011)). The role of this molecule in reproductive isolation obviously needs further experimentation.

The reduction in fruit set and well‐developed embryos following interspecific hand pollination has been reported in other orchid genera including *Pleurothallis* and *Sophronitis* (Borba et al., 2001; Silva‐Pereira et al., 2007). In our study, fruit set and the production of presumably viable, large embryos in interform pollination treatments were significantly lower than in our intraform pollination treatments in the white form. This indicated that postpollination barriers also played a role in preventing gene flow between the two forms. However, postpollination barriers between the two forms of *S. sinensis* sl were slightly weaker (especially in pollinia–pistil interactions) and more asymmetrical at the stage of fruit production. This response was asymmetrical in our white form as it produced fewer fruits compared to the pink. However, we must conclude that both asymmetric degrees were weaker than the average value for postpollination barriers (50%) as summarized by Lowry, Modliszewski et al. (2008) and Lowry, Rockwood et al. (2008) in many other plant species. In the deceptive orchids of the Mediterranean basin, the isolation barrier for fruit production was far stronger (75.4%) for 122 species of food‐deceptive orchids displaying significant asymmetry (Scopece et al., 2007). Based on embryonic development, 10 of the 38 food‐deceptive species pairs and three of the 27 sexually deceptive species pairs showed significant asymmetry (Scopece et al., 2007). Additionally, Jacquemyn, Brys, Cammue, Honnay, and Lievens (2011) found that hybridization between *Orchis purpurea* and *O. anthropophora* showed strong asymmetries both in fruit set (64.25%) and seed viability (32.94%), while *O. militaris* × *O. purpurea* showed strong asymmetries in seed viability (44.88%) with weak asymmetries in fruit set (16.37%).

In this study, prepollination barriers in both forms were stronger than postpollination barriers. In fact, the postpollination barriers were especially weak in the pink form. Cozzolino, D'Emerico, and Widmer (2004) also predicted that species with more specialized pollination systems would have stronger prepollination‐isolating barriers but weaker postpollination modes of isolation. The reverse should be true for closely related taxa sharing generalized pollination systems (Kephart & Theiss, 2004). Subdivision of modes of floral presentation in *S. sinensis* sl has resulted in a divergence in general bee pollination in which one form is now more dependent on small, diverse, solitary bees while the second depends on larger eusocial apids representing only two genera in the same family (Apidae). The two forms of *S. sinensis* sl now employ two extremes in the same bee guild but the segregation of pollinators remains incomplete for the pink form which continues to be visited by a few of the larger apids. The two color forms occupy distinct microhabitats. The white form generally distributed in wet meadows, while the pink usually grows at the edges of forest or sandy land (Tao et al. *unpublished data*). Both color forms have few opportunities to grow at close proximities to each other. Thus, the strength of pollinator isolation between white and pink color forms of *Spiranthes sinensis* would be significantly undervalued. However, this incomplete mode of isolation appears sufficient if we interpret intermediates as uncommon, recurrent hybrids. There are probably additional postpollination barriers restricting of recombination.

### 4.3 Evolutionary implications

While we can conclude that the three color forms of *S. sinensis* at our sites do not represent a panmictic and unbalanced polymorphism (sensu Futuyma, 2013), there are at least three possible hypotheses to interpret the evolutionary status of these forms. First, we consider the earlier interpretation of Mehra and Kashyap (1986) that the white and pink forms are separate species segregated on the basis of flower color, chromosome number, and elevation. Unexpectedly, our Himalayan populations did not show two different chromosome numbers, and the two dominant forms intermixed freely at different sites and elevations. At present, the only major taxonomic traits that could separate our pink and white forms are flower color and scent. Changing their status to separate species, or even varieties, seems presumptuous based on the evidence above. Yes, a number of orchid taxa have been segregated using relatively few characters, and this taxonomic replication may account, in part, for the sheer size of the family but why replicate it here? Separating orchid species or varieties, primarily on the bases of color patterns or even variations in labellum margins and size, has led to considerable taxonomic confusion leading to the proliferation of often hundreds of synonyms in some genera (e.g., *Ophrys*) as documented by Pedersen and Faurholdt (2007). While pollinator guilds in our two forms showed considerable segregation two varieties of *Cypripedium parviflorum* (var. *parviflorum* and var. *pubescens*) often overlap in mesic, North American forests and their pollinator guilds may also overlap broadly (Edens‐Meier et al., 2018).

A second interpretation is that pink and white *S. sinensis* represent two, discrete ecotypes representing locally adapted lineages. When they are sympatric, as a consequence of habitat intergradation, some interecotypic crosses must occur due to an infrequent copollinator (*Bombus friseanus*) visiting the pink form. Fruit and/or seed set decline presuming there is an intermediate, optimal outcrossing distance as proposed by Hufford, Krauss, and Veneklass (2012) as in *Stylidium hispidum* (Stylidiaceae). This could apply to our populations of *S. sinensis* as both forms showed a comparative absence of prezygotic self‐incompatibility. Seed set declines following interecotypic crosses cannot be blamed on both forms sharing S alleles. This would explain postzygotic RI after forms are crossed and it is a common and ongoing concern in conservation genetics (Frankham, Ballou, & Briscoe, 2002). As outbreeding depression has been identified experimentally in a number of seed plants (Waser & WIlliams, 2001), it should be considered here if there is a future attempt to restore populations following poaching (Bernhardt et al., 2017). Future tests must also involve the use of genetic markers (Hufford et al., 2012) absent in this study.

Third, we prefer an emphasis on evolutionary processes instead of taxonomic/ecological labels. Considering the evidence, we note that floral attractants (color and scent) appear sufficient to establish significant frequencies of prezygotic, reproductive isolation when the two forms are sympatric and coblooming. While postpollination barriers are weaker (see above), they do exist. Considering the case with which modern horticulture has produced both interspecific and intergeneric hybrids in lineages within the Orchidaceae (Dressler, 1981; Tremblay et al., 2005), this trend is predictable. Consequently, we may also regard our populations as evidence of incipient speciation. The relative strengths of reproductive isolation barriers in these two forms of the same species are regarded as evidence of a process of divergence, at least in some Himalayan regions.

Currently, limits to field observations and experimental results do not permit a precise determination of the evolutionary status of either dominant form above. In the future, it would be relevant to compare distinctive traits (i.e., color and scent) in allopatric vs. sympatric populations to test whether these traits are more divergent under sympatric vs. allopatric distributions. For example, as mentioned above, *Apis mellifera* is a congener of *A. cerana*. This domesticated and feral honeybee forages on Australian *S. sinensis* along with a native guild of smaller, bees and all carry the pink form's pollinaria (Bernhardt and Ren, *unpublished data*; Coleman, 1933) as white forms are not found in Australia (Jones, 2006). If the two color forms are not really isolated in temperate Asia, when sympatric, they represent a discrete, intraspecific, phenotypic variation as reported in many other orchid species in a number of distantly related lineages (e.g., *Paphiopedilum*, Averyanov, Cribb, Ke‐Loc, & Tien‐Hiep, 2003; *Diuris,* Jones, 2006; *Ophrys,* Pedersen & Faurholdt, 2007; *Thelymitra,* Edens‐Meier et al., 2013).

## 5 CONCLUSIONS

Our study offers evidence of reproductive isolation between white and pink forms of bee‐pollinated *S. sinensis* sl. Several reproductive barriers were identified at the prepollination and the postpollination stages restricting interform recombination. Our results indicated that strong pollinator isolation with high floral constancy by pollinarium vectors was based, at least in part, on color and scent cues. However, these barriers appear stronger in white populations and there is a trend toward unilateral isolation. In addition, significantly lower fruit sets and a decline in the production of large embryos following interform pollination indicated that postpollination barriers also played important roles in reproductive isolation.

## CONFLICT OF INTEREST

None declared.

## AUTHOR CONTRIBUTIONS

Zhi‐Bin Tao, Zong‐Xin Ren, Peter Bernhardt, Hong Wang and De‐Zhu Li designed the study and established protocols for measurements. Zhi‐Bin Tao, Zong‐Xin Ren, Hai‐Dong Li and Huan Liang performed the experiments. Zhi‐Bin Tao, Hai‐Dong Li, and Yan‐Hui Zhao analyzed the data. Zhi‐Bin Tao, Zong‐Xin Ren,and Peter Bernhardt did additional fieldwork (collecting and euthanizing pollinators) and wrote the manuscript. All authors read and approved the final manuscript.
