# Supplementary figures and images for: Does reproductive isolation reflect the segregation of color forms in Spiranthes sinensis (Pers.) Ames complex (Orchidaceae) in the Chinese Himalayas?
Source: Ecol Evol. 2018 Apr 27;8(11):5455–69. doi: 10.1002/ece3.4067 (PMC6010815; doi:10.1002/ece3.4067)

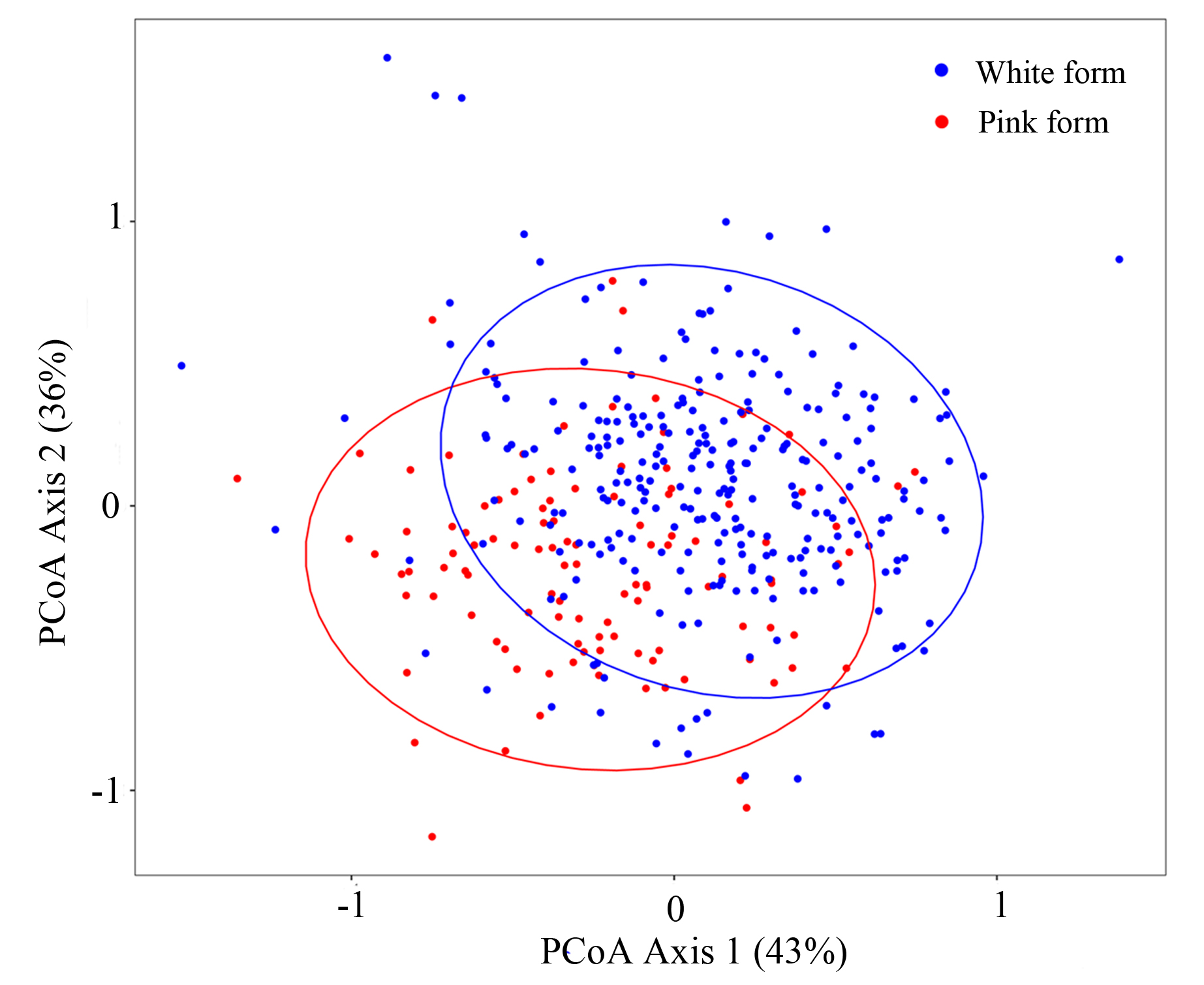

Supplement: Supplementary file 1 [file ECE3-8-5455-s001.jpg]

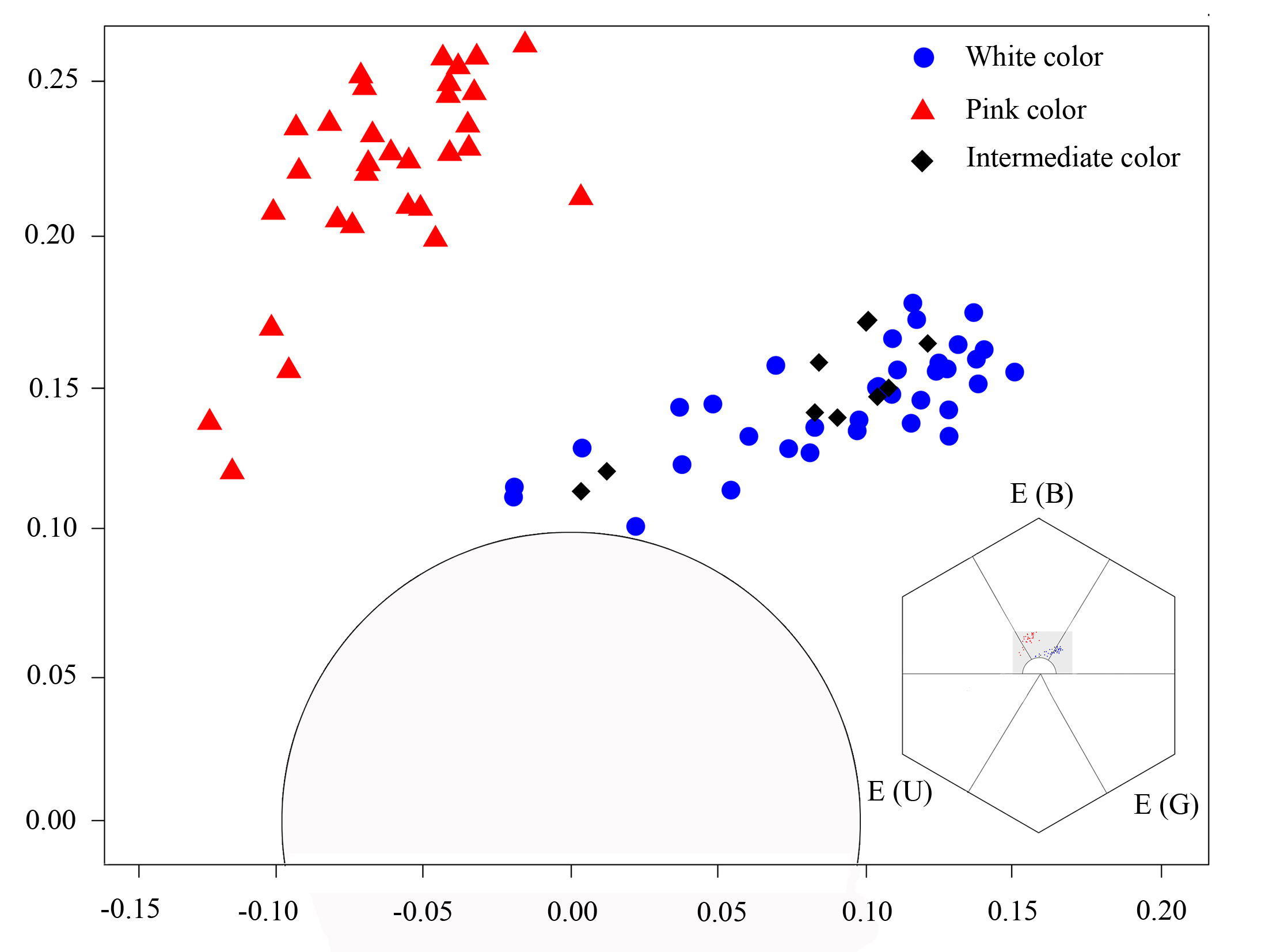

Supplement: Supplementary file 2 [file ECE3-8-5455-s002.jpg]
