# Supplementary material for: Does reproductive isolation reflect the segregation of color forms in Spiranthes sinensis (Pers.) Ames complex (Orchidaceae) in the Chinese Himalayas?
Source: Ecol Evol. 2018 Apr 27;8(11):5455–69. doi: 10.1002/ece3.4067 (PMC6010815; doi:10.1002/ece3.4067)
Supplement: Supplementary file 3 [file ECE3-8-5455-s003.doc]

Table S1**.** Comparative habitats of sympatric and allopatric populations of the three forms and the data collected in 9 sites (2014-2016). We witnessed illegal and destructive collections of these orchids in Lijiang in 2014 and 2015 and do not provide site GPS.

| Populations | Elevation  (m) | Size (m^2^) | Color morph | Habitat | Population size (total individuals) | Population density (individuals m^-2^) | Nearest distance between two forms | Data collected |
| --- | --- | --- | --- | --- | --- | --- | --- | --- |
| Yushuizhai (YSZ) | 2722 | 9089.77 | White and pink | White form: wet meadow, pink form: dry grassland | More than 2000 individuals of white form, ca. 100 individuals of pink form | White form: 2-15  Pink form: 1-5 | 20 m | Pollinator observation for white and pink form in 2014- 2016, pollen–pistil interaction for pink form in 2015. |
| Dazhuanwan (DZW) | 3021 | 13315.88 | White, pink and intermediate | White form: wet meadow, pink form: dry grassland | Ca. 400 individuals of white form, more than 150 individuals of pink form and ca. 10 individuals of intermediate form | White form: 3-11  Pink form: 2-10  Intermediate form: 0.1-1 | 1 m | Floral trait measurements for white and pink form in 2016, pollinator observations. Natural fruit set and large embryo for intermediate form in 2016 |
| Jiaochalukou (JCLK) | 3197 | 2194.74 | White | Wet meadow | Ca. 400 individuals of white form | 3-15 | – | Natural fruit sets recorded in 2016, with pollinator observations in 2016. |
| Meihuacao (MHC) | 3259 | 1932.4 | White | Wet meadow | Ca. 600 individuals of white form | 5-25 | – | Pollinator observations and breeding system in 2015 and 2016, and natural fruit sets for white form in 2016. |
| Dujuanlin (DJL) | 3298 | 2664.15 | White | Wet meadow | Ca. 500 individuals of white form | 5-20 | – | Pollinator observations and natural fruit sets in 2016. |
| Damenkou | 3286 | 2934.97 | White | Wet meadow | Ca. 200 individuals of white form | 2-10 | – | Natural fruit sets in 2016. |
| Shuiku (SK) | 3233 | 12407.74 | White, pink and intermediate | White form: wet meadow, pink form: dry grassland | More than 3000 individuals of white form, ca. 50 pink form | White form: 5-30  Pink form: 1-4  Intermediate form: 0.1-1 | 0.3 m | Phenology survey, pollen-pistil interaction, pollinator observations, breeding system in 2015 and 2016 for white and pink forms, natural fruit set and large embryo for intermediate form, and inter-color form visitation in 2016 |
| Xiaotupu (XTP) | 3265 | 3887.05 | Pink | Dry grassland | Ca. 60 individuals of red form | 1-4 | – | Pollinator observations and inter-color form visitation in 2016 |
| Shaokaodian (SKD) | 3458 | 9237.52 | White and pink | White form: wet meadow, pink form: dry grassland | More than 300 individuals of white form, ca. 200 individuals of pink form | White form: 1-15  Pink form: 1-10 | 50 m | Floral trait measurements, pollinator observation and natural rates of pollinaria removal and natural fruit set in 2016 for pink form. |
