# Supplementary material for: Does reproductive isolation reflect the segregation of color forms in Spiranthes sinensis (Pers.) Ames complex (Orchidaceae) in the Chinese Himalayas?
Source: Ecol Evol. 2018 Apr 27;8(11):5455–69. doi: 10.1002/ece3.4067 (PMC6010815; doi:10.1002/ece3.4067)
Supplement: Supplementary file 4 [file ECE3-8-5455-s004.doc]

Table S2. Voucher and GenBank accession numbers for samples used in the phylogenetic study of *Spiranthes sinensis* sl and related taxa; “NA” indicates information is not available for this category. * represents sequences of this species obtained in this study (sequences of remaining taxa were downloaded from GenBank).

| Species  names | Color morph | Sample ID | Locality | GenBank Accessions | |
| --- | --- | --- | --- | --- | --- |
|  |  |  |  | trnS-G | matK |
| *S. sinensis* | NA | 29 | Malaysia | KM283584 | KM262487 |
| *S. sinensis* | NA | Si17 | Yunnan, China, | KM283585 | KM262488 |
| *S. aestivalis* | NA | Sa58 | London, UK | KM283593 | KM262491 |
| *S. aestivalis* | NA | Sa59 | Switzerland | KM283594 | KM262492 |
| *S. spiralis* | NA | Sp51 | UK | KM283595 | KM262490 |
| *S. tuberosa* | NA | 26b | South Carolina, American | KM283567 | KM262465 |
| *S. tuberosa* | NA | 26c | West virginia, American | KM283568 | KM262466 |
| *S. tuberosa* | NA | 26e | Florida, America | KM283569 | KM262467 |
| *S. tuberosa* | NA | 26f | South Carolina, American | KM283570 | KM262468 |
| *S. tuberosa* | NA | 26g | Florida, America | KM283571 | KM262469 |
| *S. sinensis** | White | S1 | DZW, Lijiang, Yunan, China | MH036729 | MH036715 |
| *S. sinensis** | White | S2 | DZW, Lijiang, Yunan, China | MH036730 | MH036716 |
| *S. sinensis** | White | S3 | SK, Lijiang, Yunan, China | MH036731 | MH036717 |
| *S. sinensis** | Pink | S4 | SKD, Lijiang, Yunan, China | MH036732 | MH036718 |
| *S. sinensis** | Pink | S5 | SKD, Lijiang, Yunan, China | MH036733 | MH036719 |
| *S. sinensis** | Pink | S6 | SK, Lijiang, Yunan, China | MH036734 | MH036720 |
| *S. sinensis** | Intermediate | S7 | SK, Lijiang, Yunan, China | MH036735 | MH036721 |
| *S. sinensis** | Intermediate | S8 | SK, Lijiang, Yunan, China | MH036736 | MH036722 |
| *S. sinensis** | Intermediate | S9 | SK, Lijiang, Yunan, China | MH036737 | MH036723 |
| *S. sinensis**(*S. australis*) | Pink | S10 | Kulura, NSW, Australia | MH036738 | MH036724 |
| *S. sinensis**(*S. australis*) | Pink | S11 | Kulura, NSW, Australia | MH036739 | MH036725 |
| *S. sinensis**(*S. australis*) | Pink | S12 | Kulura, NSW, Australia | MH036740 | MH036726 |
| *S. sinensis** | Pink | S13 | Ninglang, Yunan, China | MH036741 | MH036727 |
| *S. sinensis** | Pink | S14 | Ninglang, Yunan, China | MH036742 | MH036728 |
