# Supplementary material for: Does reproductive isolation reflect the segregation of color forms in Spiranthes sinensis (Pers.) Ames complex (Orchidaceae) in the Chinese Himalayas?
Source: Ecol Evol. 2018 Apr 27;8(11):5455–69. doi: 10.1002/ece3.4067 (PMC6010815; doi:10.1002/ece3.4067)
Supplement: Supplementary file 5 [file ECE3-8-5455-s005.doc]

Table S3**.** Total reproductive isolation and the absolute and relative contribution to total reproductive isolation between white and pink forms.

| Isolating barriers | Components of RI | | Asymmetry of each barrier | Absolute contributions to RI_total_ | | Relative contribution to RI_total_ | |
| --- | --- | --- | --- | --- | --- | --- | --- |
|  | White | Pink |  | White | Pink | White | Pink |
| Pre-mating barriers |  |  |  |  |  |  |  |
| Phenology | 0.14583 | 0.10870 | 0.03714 | 0.14583 | 0.10870 | 0.15059 | 0.16286 |
| Pollinator | 0.87768 | 0.52381 | 0.35387 | 0.74874 | 0.45560 | 0.77315 | 0.68262 |
| Total RI_pre-pollination_ | — | — | — | 0.89457 | 0.56429 | 0.92373 | 0.84547 |
| Postpollination barriers |  |  |  |  |  |  |  |
| Pollen-pistil interaction | -0.02736 | 0.09239 | 0.11974 | -0.00560 | 0.05985 | -0.00578 | 0.08967 |
| Fruit set | 0.39453 | 0.05864 | 0.33589 | 0.06126 | 0.03453 | 0.06325 | 0.05174 |
| Embryo mortality | 0.22819 | 0.01563 | 0.21256 | 0.01820 | 0.00876 | 0.01879 | 0.01312 |
| Total RI_post-pollinaiton_ | — | — | — | 0.07386 | 0.10314 | 0.07627 | 0.15453 |
| Total RI | — | — | — | 0.96843 | 0.66742 | 1 | 1 |
